# Sorafenib versus Lenvatinib Causes Stronger Oxidative Damage to Membrane Lipids in Noncancerous Tissues of the Thyroid, Liver, and Kidney: Effective Protection by Melatonin and Indole-3-Propionic Acid

**DOI:** 10.3390/biomedicines10112890

**Published:** 2022-11-11

**Authors:** Jan Stępniak, Joanna Krawczyk-Lipiec, Andrzej Lewiński, Małgorzata Karbownik-Lewińska

**Affiliations:** 1 Department of Oncological Endocrinology, Medical University of Lodz, 90-752 Lodz, Poland; 2 Polish Mother’s Memorial Hospital—Research Institute, 93-338 Lodz, Poland; 3 Department of Endocrinology and Metabolic Diseases, Medical University of Lodz, 93-338 Lodz, Poland

**Keywords:** sorafenib, lenvatinib, lipid peroxidation, melatonin, indole-3-propionic acid

## Abstract

Sorafenib and lenvatinib are multi-targeted tyrosine kinase inhibitors which are currently approved to treat advanced hepatocellular carcinoma, renal cell carcinoma and radioiodine-refractory differentiated thyroid carcinoma. However this treatment is often limited due to common adverse events which may occur via oxidative stress. The study aims to compare sorafenib- and lenvatinib-induced oxidative damage to membrane lipids (lipid peroxidation, LPO) in homogenates of porcine noncancerous tissues of the thyroid, the liver, and the kidney and to check if it can be prevented by antioxidants melatonin and indole-3-propionic acid (IPA). Homogenates of individual tissues were incubated in the presence of sorafenib or lenvatinib (1 mM, 100 µM, 10 µM, 1 µM, 100 nM, 10 nM, 1 nM, 100 pM) together with/without melatonin (5.0 mM) or IPA (5.0 mM). The concentration of malondialdehyde + 4-hydroxyalkenals, as the LPO index, was measured spectrophotometrically. The incubation of tissue homogenates with sorafenib resulted in a concentration-dependent increase in LPO (statistically significant for concentrations of 1mM and 100 µM in the thyroid and the liver, and of 1 mM, 100 µM, and 10 µM in the kidney). The incubation of thyroid homogenates with lenvatinib did not change LPO level. In case of the liver and the kidney, lenvatinib increased LPO but only in its highest concentration of 1 mM. Melatonin and IPA reduced completely (to the level of control) sorafenib- and lenvatinib-induced LPO in all examined tissues regardless of the drug concentration. In conclusion, sorafenib comparing to lenvatinib is a stronger damaging agent of membrane lipids in noncancerous tissues of the thyroid, the liver, and the kidney. The antioxidants melatonin and IPA can be considered to be used in co-treatment with sorafenib and lenvatinib to prevent their undesirable toxicity occurring via oxidative stress.

## 1. Introduction

Over the last two decades considerable progress has been made in developing new anticancer medications. One group of these agents are small-molecule kinase inhibitors which, due to their advantages such as high selectivity, high efficacy, low side effects and ease of preparation, found application as the first-line drug in the treatment of a number of cancers. There are several dozen kinase inhibitors, sorafenib and lenvatinib included, clinically approved around the world [1].

Both sorafenib and lenvatinib, belonging to multi-targeted tyrosine kinase inhibitors, have shown in vitro and in vivo efficacy in a broad range of malignances, including renal cell, hepatocellular, breast, pancreas, ovarian and colon cancer [2,3,4]. They are currently approved for the treatment of advanced hepatocellular carcinoma (HCC), renal cell carcinoma (RCC) and radioiodine-refractory differentiated thyroid carcinoma (RR-DTC) [5,6]. Therefore, the liver, kidney, and thyroid tissues, however unchanged by the neoplastic process, were used in the present study to check if cell lipid membranes of these three “normal” tissues are affected by sorafenib or lenvatinib.

Sorafenib is a bis-aryl urea chemical compound that has anticancer action based on inhibition of angiogenesis (tumor blood supply) and tumor cell proliferation (tumor growth) through the blockage of Raf/MEK/ERK and JAK/STAT signaling pathways and through the inhibition of multiple tyrosine kinases of such receptors as vascular endothelial growth factor receptor (VEGFR)-2, VEGFR-3, platelet-derived growth factor receptor beta (PDGFRβ), FMS-like tyrosine kinase 3 (FLT3), rearranged during transfection receptor (RET) and tyrosine-kinase receptor (KIT) [7].

Lenvatinib is a member of the class of quinoline carboxiamides and is an anti-tumor drug with a predominant anti-angiogenic effect. It acts by inhibiting the kinase activities of VEGFR1-3, fibroblast growth factor receptors family (FGFR) 1-4, platelet-derived growth factor receptor alpha (PDGFRα), KIT, and RET [3]. Lenvatinib treatment of HCC and RR-DTC is associated with a longer progression-free survival than treatment with sorafenib [8,9].

Although sorafenib and lenvatinib are currently one of the few that are effective therapy in advanced HCC and have been documented to significantly improve survival of patients diagnosed with RCC and RR-DTC the treatment is often limited due to common adverse events. The side effects such as palmoplantar erythrodysesthesia (hand-foot syndrome), hematological toxicities (i.e., anemia, leukopenia, neutropenia, lymphopenia), diarrhea, nausea, vomiting and fatigue are reported in most patients [10,11,12]. In some cases they may mandate for reduction of anticancer agent dose or even for discontinuation of therapy. 

Underlying mechanisms of adverse events of sorafenib and lenvatinib treatment and of their unwanted toxicities are not completely understood. As they are multiple tyrosine kinases inhibitors, they can interact with many signaling pathways on various levels, therefore one main mechanism of toxicity is difficult, if not impossible, to determine. However, it has been demonstrated in some studies that one of the mechanisms of this toxicity may be associated with reactive oxygen species (ROS) generation [13,14]. It has been also confirmed that sorafenib used in chronic treatment induces liver toxicity by activation of oxidative stress [15]. Moreover, evidence exists that antioxidant enzymes such as catalase and glutathione peroxidase (GPX) have diminished activities in sorafenib-treated animals [15]. All of these observations directly indicate that both sorafenib and lenvatinib are strongly involved in the oxidative processes in the cell. 

Taking the above into consideration, a need arises for studies concerning the impact (and its mechanisms) of these drugs on tissues (or their parts) not affected by the neoplastic process. In addition, to identify possible methods to prevent unwanted toxicity caused by sorafenib/lenvatinib is important to improve treatment efficiency and quality of life in cancer patients.

To offset the negative oxidative effects of tyrosine kinase inhibitors, the use of endogenous antioxidants may be helpful. One of the best known endogenous antioxidants and free radical scavengers is N-acetyl-5-methoxytryptamine (melatonin) [16]. It is an indole hormone synthesized from tryptophan and mainly secreted by the pineal gland. Similar in chemical structure to melatonin (possessing a heterocyclic aromatic ring) is indole-3-propionic acid (IPA), which is a deamination product of tryptophan. In the human body, it is produced by intestinal bacteria and closely associated with diet [17]. These two indole substances possess significant antioxidative properties, which was confirmed in numerous experimental or clinical studies [18,19,20].

The aim of this study was to compare pro-oxidative effects of sorafenib and lenvatinib on cell lipid membranes in homogenates of three porcine noncancerous tissues, i.e., the thyroid, liver, and kidney (which, in case of malignant transformation, respond to these anticancer medications) and to check the potential protective (antioxidative) action of melatonin and IPA against damaging effects of these two tyrosine kinase inhibitors. Pro-/antioxidative effects were evaluated by measurement of lipid peroxidation (LPO), being an index of oxidative damage to membrane lipids.

## 2. Materials and Methods

### 2.1. Ethical Considerations

In accordance with the Polish Act on the Protection of Animals Used for Scientific or Educational Purposes from 15 January 2015 (which implements Directive 2010/63/EU of the European Parliament and the Council of 22 September 2010 on the protection of animals used for scientific purposes)—the use of animals to collect organs or tissues does not require the approval of the Local Ethics Committee. These animals are only subjected to registration by the center in which the organs or tissues were taken. Additionally, we did not use experimental animals; instead, porcine tissues were collected from animals at a slaughterhouse during the routine process of slaughter carried out for consumption.

### 2.2. Chemicals

Melatonin, indole-3-propionic acid and lenvatinib were purchased from Sigma (St. Louis, MO, USA). Sorafenib was purchased from LC Laboratories (Woburn, MA, USA). The LPO-586 kit for LPO was obtained from Enzo Life Science (Farmingdale, NY, USA). All the used chemicals were of analytical grade and came from commercial sources. 

### 2.3. Tissue Collection

Porcine tissues were collected from pigs slaughtered at the local slaughterhouse. Animals were treated according to the European Community Council Regulation (CE1099/2009) concerning protection of animals at the time of killing. All animals were sexually mature as determined by age (8–9 months) and body mass [116 ± 2.9 (SD) kg]. Immediately after the slaughter, the tissues, i.e., the thyroid, the liver and the kidney, were collected, frozen on solid CO_2_, and stored at −80 °C till experimental procedure. Each experiment was repeated three times.

### 2.4. Incubation of Tissue Homogenates

Porcine tissues (the thyroid, the liver and the kidney) were homogenized in ice cold 20 mM Tris-HCl buffer (pH 7.4) (10%, *w*/*v*) and then incubated for 30 min at 37 °C in the presence of sorafenib (1 mM, 100 µM, 10 µM, 1 µM, 100 nM, 10 nM, 1 nM, 100 pM) or lenvatinib (1 mM, 100 µM, 10 µM, 1 µM, 100 nM, 10 nM, 1 nM, 100 pM) prepared in dimethyl sulfoxide (DMSO) (final conc. 1%) without or with addition of melatonin or indole-3-propionic acid (both in concentration of 5.0 mM). The reactions were stopped by cooling the samples on ice.

### 2.5. Assay of Lipid Peroxidation

The concentrations of malondialdehyde + 4-hydroxyalkenals (MDA + 4-HDA), as an index of lipid peroxidation, were measured in homogenates with the LPO-586 kit. The homogenates were centrifuged at 5000× *g* for 10 min at 4 °C. After obtaining supernatant, each experiment was carried out in duplicate. The supernatant (200 μL) was mixed with 650 μL of a methanol:acetonitrile (1:3, *v*/*v*) solution, containing a chromogenic reagent, i.e., N-methyl-2-phenylindole, and vortexed. Following the addition of 150 μL of methanesulfonic acid (15.4 M), the incubation was carried out at 45 °C for 40 min. The reaction between MDA + 4-HDA and N-methyl-2-phenylindole yields a chromophore, which is spectrophotometrically measurable at an absorbance of 586 nm, using a solution of 10 mM 4-hydroxynonenal as the standard. The level of lipid peroxidation is expressed as the amount of MDA + 4-HDA (nmol) per mg protein. Protein was measured using Bradford’s method, with bovine albumin as the standard [21]. 

### 2.6. Statistical Analyses

The data were statistically analyzed, using a one-way analysis of variance (ANOVA), followed by the Student–Neuman–Keuls’ test, or using an unpaired *t*-test. Statistical significance was determined at the level of *p* < 0.05. Results are presented as means ± SE.

## 3. Results

**Figure 1:**
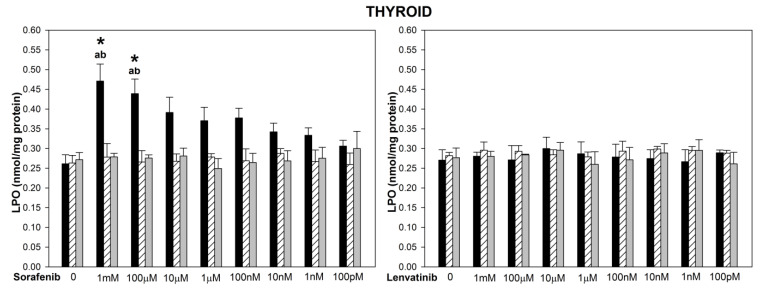
Lipid peroxidation (LPO), measured as MDA + 4-HDA level, in porcine thyroid homogenates, incubated in the presence of sorafenib (1 mM, 100 µM, 10 µM, 1 µM, 100 nM, 10 nM, 1 nM, 100 pM) alone or lenvatinib (1 mM, 100 µM, 10 µM, 1 µM, 100 nM, 10 nM, 1 nM, 100 pM) alone (black bars), or sorafenib/lenvatinib together with melatonin (5.0 mM) (striped bars) or together with indole-3-propionic acid (5.0 mM) (grey bars). LPO level is expressed in nmol/mg protein. Data are presented as mean ± SE (error bars). ^a^ *p* < 0.05 vs. control (without any substance); ^b^ *p* < 0.05 vs. respective concentration of sorafenib with melatonin (5.0 mM) or with indole-3-propionic acid (5.0 mM); * *p* < 0.05 vs. lenvatinib in the same concentration.

**Figure 2:**
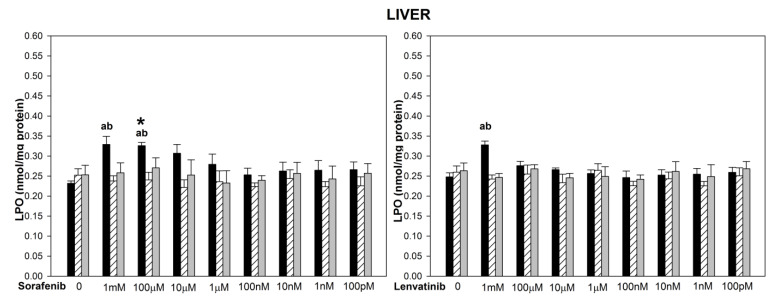
Lipid peroxidation (LPO), measured as MDA + 4-HDA level, in porcine liver homogenates, incubated in the presence of sorafenib (1 mM, 100 µM, 10 µM, 1 µM, 100 nM, 10 nM, 1 nM, 100 pM) alone or lenvatinib (1 mM, 100 µM, 10 µM, 1 µM, 100 nM, 10 nM, 1 nM, 100 pM) alone (black bars), or sorafenib/lenvatinib together with melatonin (5.0 mM) (striped bars) or together with indole-3-propionic acid (5.0 mM) (grey bars). LPO level is expressed in nmol/mg protein. Data are presented as mean ± SE (error bars). ^a^ *p* <0.05 vs. control (without any substance); ^b^ *p* < 0.05 vs. respective concentration of sorafenib with melatonin (5.0 mM) or with indole-3-propionic acid (5.0 mM); * *p*< 0.05 vs. lenvatinib in the same concentration.

**Figure 3:**
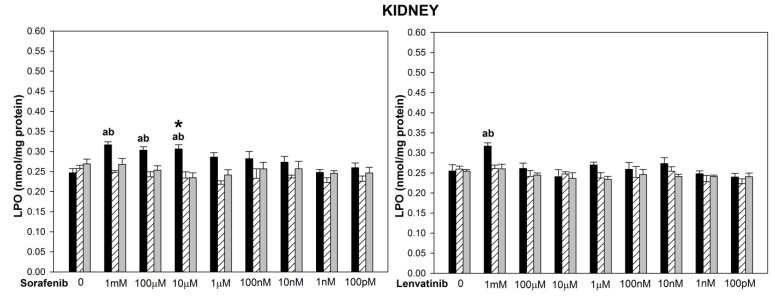
Lipid peroxidation (LPO), measured as MDA + 4 − HDA level, in porcine kidney homogenates, incubated in the presence of sorafenib (**A**) (1 mM, 100 µM, 10 µM, 1 µM, 100 nM, 10 nM, 1 nM, 100 pM) or lenvatinib (**B**) (1 mM, 100 µM, 10 µM, 1µM, 100 nM, 10 nM, 1 nM, 100 pM) alone (black bars), or sorafenib/lenvatinib together with melatonin (5.0 mM) (striped bars) or together with indole-3-propionic acid (5.0 mM) (grey bars). LPO level is expressed in nmol/mg protein. Data are presented as mean ± SE (error bars). ^a^ *p* < 0.05 vs. control (without any substance); ^b^ *p* < 0.05 vs. respective concentration of sorafenib with melatonin (5.0 mM) or with indole-3-propionic acid (5.0 mM); * *p* < 0.05 vs. lenvatinib in the same concentration.

The basal LPO levels did not differ significantly among three examined tissues (Figure 1, Figure 2 and Figure 3). The incubation of thyroid, liver and kidney homogenates in the presence of sorafenib resulted in a concentration-dependent increase in LPO level (statistically significant for concentrations of 1 mM and 100 µM in the thyroid and the liver, and of 1 mM, 100 µM, and 10 µM in the kidney) (Figure 1, Figure 2 and Figure 3 left graphs). Lipid peroxidation induced by sorafenib did not differ significantly between the thyroid and the liver (for example, for sorafenib concentration of 1 mM—*p =* 0.099) (Figure 1 and Figure 2 left graphs), as well as between the thyroid and the kidney (for example, for sorafenib concentration of 1 mM—*p =* 0.094) (Figure 1 and Figure 3 left graphs).

The incubation of thyroid homogenates in the presence of lenvatinib did not change the LPO level (Figure 1 right graph). In the case of the liver and the kidney, lenvatinib did increase LPO but only in its highest used concentration of 1 mM (Figure 2 and Figure 3 right graphs). Levels of LPO obtained after lenvatinib (1 mM) treatment did not differ between the liver and the kidney, but they both were higher than in the thyroid (*p =* 0.027 and *p =* 0.045, respectively) (Figure 1, Figure 2 and Figure 3 right graphs).

When comparing LPO level obtained in response to sorafenib versus lenvatinib, it was significantly higher after sorafenib treatment in the thyroid (for concentrations of 1 mM and 100 µM) (Figure 1), in the liver (for the concentration of 100 µM) (Figure 2), and in the kidney (for the concentration of 10 µM) (Figure 3).

Melatonin (5.0 mM) and indole-3-propionic acid (5.0 mM) reduced completely (to the level of control) sorafenib- or lenvatinib-induced lipid peroxidation in all examined tissues regardless of the concentration of these drugs (Figure 1, Figure 2 and Figure 3). Both melatonin and indole-3-propionic acid did not change the basal LPO level in all examined tissues (Figure 1, Figure 2 and Figure 3).

## 4. Discussion

As was mentioned in the Introduction, both sorafenib and lenvatinib do constitute an effective first-line medical therapy for a number of malignances, however, in most cases they cause numerous adverse effects that can be related to oxidative stress generated by these drugs. Therefore, it is of interest to check which of these medications is a stronger prooxidant with relation to tissues (or their parts) not affected by the neoplastic process. Additionally, it is important for effective therapy to find ways to prevent unfavorable oxidative damage due to anticancer therapy.

Whereas we have chosen in the present study the tissues that can develop malignancies with confirmed sensitivity to sorafenib/lenvatinib treatment, it should stressed that the assays were performed with the use of only noncancerous (“normal”) tissues. Therefore, our results should be interpreted in relation to rather “healthy” (not affected by malignant process) compartments of the organism. Thus, pro-oxidative action of sorafenib/lenvatinib observed in the present study may theoretically constitute a part of mechanisms (probably one of numerous) of adverse effects of these medications. Regarding the influence of these kinase inhibitors on oxidative processes in cancerous tissues, the present study does not constitute a good experimental model. 

Our study is limited to measurement of oxidative damage to only one biological macromolecule, i.e., membrane lipids. With relation to this process sorafenib was found to be a stronger damaging agent comparing to lenvatinib. This is an interesting observation considering that peroxidation of membrane lipids may play a significant role in the mechanism of anticancer action of both drugs. According to the recent studies sorafenib and lenvatinib are also potent inducers of ferroptosis [15,22]—an iron-dependent form of programmed cell death. In case of HCC, ferroptosis is postulated to be one of the main mechanisms responsible for anticancer action of these drugs [15,22,23]. The most important hallmark of ferroptosis is lipid peroxidation being a process driven by free radical produced in iron-dependent Fenton reaction [24]. During this reaction hydrogen peroxide (H_2_O_2_) interacts with iron (II) to form hydroxyl radical (^•^OH). The ^•^OH, being most dangerous reactive oxygen species, reacts—at a diffusion-controlled rate—with almost all types of biological macromolecules (i.e., carbohydrates, nucleic acids, lipids and amino acids), forming products which contribute to further damage of biological structures [25]. Degradation of lipids (especially polyunsaturated fatty acids) and cholesterol in cytoplasmic or organelle membranes leads to structural changes, impairing cellular functions and consequently cell death. It has been found that level of malondialdehyde (MDA) and 4-hydroxynonenal (HNE) (two major by-products of lipid peroxidation, which were measured in the present study) is increased during ferroptosis [26]. Mechanisms responsible for pro-ferroptotic actions of sorafenib and lenvatinib are still not fully understood. However, it is postulated that in case of sorafenib, an underlying mechanism of ferroptosis induction is based on cystine/glutamate antiporter (x_c_^−^) inhibition. System x_c_^−^ is an amino acid antiporter that participates in the exchange of extracellular l-cystine and intracellular l-glutamate across the cellular plasma membrane. Inhibition of this system causes depletion of intracellular glutathione (GSH), a reducing substrate used by glutathione peroxidase 4 (GPX4), which is a key regulator of lipid peroxidation [27]. The decrease in the activity of GPX4 results in the accumulation of membrane lipid peroxides, oxidative damage to lipids, and subsequently in ferroptosis. In case of lenvatinib, it has been demonstrated that this drug induces ferroptosis—similarly to sorafenib—by suppressing x_c_^−^ system, and this suppression is the result of FGFR4 inhibition [22]. As in our study sorafenib has shown stronger than lenvatinib pro-oxidative effect on cell membrane lipids one might assume that sorafenib is a stronger stimulant of the ferroptosis process and in consequence may show a stronger anti-cancer effect. However, our results obtained with the use of normal noncancerous tissue may not be extrapolated to conditions associated with malignant process.

Regarding clinical observations, in a randomized clinical trials comparing anti-cancer effectiveness it has been demonstrated that treatment of RR-DTC with lenvanitib was associated with a longer progression-free survival compared with sorafenib [8,28]. In case of treatment of HCC, lenvatinib showed comparable overall survival and progression-free survival but longer time to progression and better tumor response compared to sorafenib [29,30]. However, treatment of both, i.e., RR-DTC and HCC, with lenvatinib was associated with more frequently occurring severe adverse effects, especially hypertension and proteinuria [28,29,30]. It should be noted that generally adverse events lead to dose reduction or discontinuation of treatment more frequently in cases of lenvatinib than ofsorafenib [9]. 

Taking into account these clinical observations, it is difficult to use findings of the present study to explain differences between sorafenib vs. lenvatinib regarding their anticancer effectiveness or side effects. Perhaps it can be proposed that weaker oxidative damage to macromolecules in healthy tissues due to lenvatinib observed in the present study may contribute to its higher efficacy in the treatment. At the same time more commonly occurring undesired effects of lenvatinib therapy could result from other than oxidative mechanisms. Additionally, the results of the present in vitro study cannot be directly extrapolated to conditions in the organism of cancer patients.

In the present study, we have also observed that both melatonin and IPA revealed antioxidant properties and completely reduced lipid peroxidation caused by sorafenib and lenvatinib. From these two antioxidative substances, melatonin mechanisms of action are better understood. As an amphiphilic compound (i.e., combining both hydrophilic and hydrophobic properties caused by its O-methyl and N-acetyl residues), melatonin can diffuse and easily cross all cell barriers [31,32]. Because of its electron-rich aromatic ring system this indoleamine can easily function as an electron donor and can directly neutralize toxic free radicals more efficiently compared with other classical antioxidants (it is twice as active as vitamin E, which is the reference substance in the field) [16]. Moreover, melatonin can stimulate the expression of endogenous antioxidant enzymes such as superoxide dismutase, glutathione reductase, GPX, and catalase [16]. Melatonin can also increase the efficiency of the mitochondrial electron transport chain which reduces electron leakage and, consequently, reduces the generation of free radicals [33,34]. It has been also observed that melatonin can suppress ferroptosis through activation of nuclear factor erythroid 2-related factor 2 (Nrf2)/heme oxygenase-1 (HO-1) pathway [35,36] and also by direct ferrous ion chelation [37,38]. 

It should be stressed that melatonin can also act as a conditional pro-oxidant (acting only under special strictly defined conditions such as, e.g., cancerogenesis), especially in tumor cells and it is able to promote ROS generation at pharmacological concentrations [39,40]. However, this action of melatonin has been well-documented exclusively under in vitro conditions [41]. Beside this anticancer pro-oxidative action, which should be treated as a favorable effect, melatonin is able to suppress tumor growth, proliferation, and metastasis by mechanisms, such as induction of apoptosis, suppression of angiogenesis and tumor cell repopulation and stimulation of immune system cells [42]. Especially the last phenomenon, i.e., promotion of immune system response, may have special significance as immunotherapy is a promising new strategy to treat cancer. For example, in case of HCC therapy with immune checkpoint inhibitors yielded better overall and progression-free survival outcomes [43].

Melatonin also shows a wide range of the synergistic effects with anticancer drugs (including sorafenib) or radiotherapy [44,45,46,47,48,49]. Studies so far have demonstrated that melatonin can reinforce antitumor effect of vincristine and ifosfamide on Ewing sarcoma cells [43] as well as drugs, such as doxorubicin, cisplatin, and 5-fluorouracil on HeLa cells [44]. These reinforce antitumor effects of chemotherapy drugs are mediated through enhancement of apoptosis via, respectively, upregulation of extrinsic or intrinsic apoptosis pathway due to reactive oxygen species (ROS) overproduction. Melatonin can also improve the efficiency of temozolomide therapy by downregulation of the expression and function of the ABC transporter ABCG2/BCRP in malignant gliomas [46].

There are also numerous studies reporting synergistic effects of melatonin cotreatment with sorafenib. It has been shown that melatonin enhances the sensitivity of cancer cells to sorafenib and cytotoxicity induced by this drug [47,48]. In our study melatonin has shown antioxidant protective properties against lipid peroxidation caused by sorafenib and lenvatinib. Such an observation is promising in the context of potential protective effects of melatonin against at least some adverse effects induced by these tyrosine kinase inhibitors. This may constitute a rationale for the future research and for consideration to extend the use of melatonin in anticancer therapy.

However, it should be stressed that using such potent antioxidant during chemotherapy may paradoxically lead to the possibility of tumor protection and reduction of survival prognosis via different mechanisms. For example, through excessively reducing ROS formation and ability to chelate transition metals [50,51], melatonin may serve as a barrier to ferroptosis and induce sorafenib and lenvatinib resistance. Therefore, more studies are needed to address concerns regarding these relationships. Nevertheless, melatonin can be regarded as a highly promising agent to be used in pharmacological treatment [49,52].

In the present study we have also observed antioxidative protective effects of IPA. This endogenous antioxidant, chemically similar to melatonin, can be also considered in cotreatment with anticancer agents, such as sorafenib or lenvatinib, although with similar caution.

Our study has, of course, some limitations. The most important is that we studied effects of sorafenib/lenvatinib only on noncancerous tissues and, therefore, the present results are not helpful to answer the question how these two kinase inhibitors affect oxidative processes in cancerous tissues. Additionally, we tested only these tissues which, in case of malignant process, are sensitive to kinase inhibitors; however, other different tissues can also be assessed in future studies for oxidative processes in response to sorafenib/lenvatinib. The important limitation is that our experimental model is an in vitro study with the use of tissue homogenates and, therefore, our results may not be directly extrapolated into in vivo conditions, especially into human organisms. Regarding clinical studies, it can be planned in the future just to measure LPO and possibly other markers of oxidative stress in blood serum collected from patients with malignancies during treatment with kinase inhibitors. Another limitation is that in the present study, we measured only one index of oxidative damage to macromolecules, i.e., oxidative damage to membrane lipids (LPO); although different macromolecules react usually in the same way to oxidative abuse caused by a given factor, it would be worth checking in the next studies how sorafenib and lenvatinib affect protein and, especially, DNA oxidative damage.

In conclusion, sorafenib comparing to lenvatinib is a stronger damaging agent of membrane lipids in noncancerous tissues of the thyroid, the liver, and the kidney, being the tissues that, in case of malignant transformation, are sensitive to these anticancer agents. It is difficult to conclude how these differences in pro-oxidative action between sorafenib and lenvatinib contribute to treatment effectiveness or adverse effects and therefore this issue requires further experimental studies. Because melatonin and IPA revealed antioxidant properties and completely reduced lipid peroxidation caused by sorafenib and lenvatinib, both antioxidants can be considered to be used in co-treatment with these tyrosine kinase inhibitors to prevent their undesirable toxicity occurring via oxidative stress.

## Data Availability

The datasets used and/or analyzed during the current study are available from the corresponding author on reasonable request.
